# Bioactive Sphingolipids, Complement Cascade, and Free Hemoglobin Levels in Stable Coronary Artery Disease and Acute Myocardial Infarction

**DOI:** 10.1155/2018/2691934

**Published:** 2018-07-09

**Authors:** T. Jadczyk, K. Baranski, M. Syzdol, E. Nabialek, W. Wanha, R. Kurzelowski, M. Z. Ratajczak, M. Kucia, B. Dolegowska, M. Niewczas, J. Zejda, W. Wojakowski

**Affiliations:** ^1^Division of Cardiology and Structural Heart Diseases, Medical University of Silesia, Ziołowa 45-47, Katowice, Poland; ^2^International Clinical Research Center, St. Anne's University Hospital Brno, Brno, Czech Republic; ^3^Department of Epidemiology, Medical University of Silesia, Katowice, Poland; ^4^Stem Cell Institute at James Graham Brown Cancer Center, University of Louisville School of Medicine, Louisville, USA; ^5^Department of Laboratory Medicine, Pomeranian Medical University, Szczecin, Poland; ^6^Department of Sport, Faculty of Physical Education, University of Rzeszow, Rzeszow, Poland

## Abstract

**Background:**

Acute myocardial infarction (AMI) and coronary artery bypass graft (CABG) surgery are associated with a pathogen-free inflammatory response (sterile inflammation). Complement cascade (CC) and bioactive sphingolipids (BS) are postulated to be involved in this process.

**Aim:**

The aim of this study was to evaluate plasma levels of CC cleavage fragments (C3a, C5a, and C5b9), sphingosine (SP), sphingosine-1-phosphate (S1P), and free hemoglobin (fHb) in AMI patients treated with primary percutaneous coronary intervention (pPCI) and stable coronary artery disease (SCAD) undergoing CABG.

**Patients and Methods:**

The study enrolled 37 subjects (27 male) including 22 AMI patients, 7 CABG patients, and 8 healthy individuals as the control group (CTRL). In the AMI group, blood samples were collected at 5 time points (admission to hospital, 6, 12, 24, and 48 hours post pPCI) and 4 time points in the CABG group (6, 12, 24, and 48 hours post operation). SP and S1P concentrations were measured by high-performance liquid chromatography (HPLC). Analysis of C3a, C5a, and C5b9 levels was carried out using high-sensitivity ELISA and free hemoglobin by spectrophotometry.

**Results:**

The plasma levels of CC cleavage fragments (C3a and C5b9) were significantly higher, while those of SP and S1P were lower in patients undergoing CABG surgery in comparison to the AMI group. In both groups, levels of CC factors showed no significant changes within 48 hours of follow-up. Conversely, SP and S1P levels gradually decreased throughout 48 hours in the AMI group but remained stable after CABG. Moreover, the fHb concentration was significantly higher after 24 and 48 hours post pPCI compared to the corresponding postoperative time points. Additionally, the fHb concentrations increased between 12 and 48 hours after PCI in patients with AMI.

**Conclusions:**

Inflammatory response after AMI and CABG differed regarding the release of sphingolipids, free hemoglobin, and complement cascade cleavage fragments.

## 1. Introduction

According to WHO, cardiovascular diseases (CVD) are the leading cause of morbidity and mortality worldwide (17.5 million per year) [1]. The epidemiological data reflects the global trend for Europe, where coronary artery disease (CAD) with its complication in the form of acute myocardial infarction (AMI) and, in the longer-term, heart failure, accounts for 20% of all deaths among Europeans [2, 3].

Despite the significant advancement in therapeutic strategy including optimized pharmacotherapy and myocardial revascularization, the prognosis of patients with CVD remains unsatisfactory. Addressing this medical and socioeconomical challenge, a better understanding of the pathophysiology of myocardial ischemia must be achieved. Since inflammation plays the key role in coronary plaque rupture, new markers of this process such as bioactive sphingolipids (BS) and complement cascade (CC) seem to be important.

Both forms of myocardial injury—AMI and CABG procedure—trigger an intense local and systematic inflammatory response in pathogen-free form termed sterile inflammation [4]. At the molecular level, this process relies on a complex intracellular interaction network orchestrated by chemoattractant gradient of growth factors, cytokines, kinins, chemokines, BS, CC, coagulation, and fibrinolysis cascades [5–8]. Conventionally, activation of the inflammatory response is associated with adverse clinical outcomes. However, according to new research data, CC and BS play an essential role also in the myocardial repair process.

BS, including sphingosine (SP) and sphingosine-1-phosphate (S1P)[9, a biologically important class of compounds, have essential functions including regulation of cell growth, differentiation, proliferation, adhesion, migration, and apoptosis as well as inflammation and angiogenesis [10]. Erythrocytes (generating almost half of S1P concentration in blood), activated platelets, albumin, high-density lipoproteins, endothelial cells, and circulating microvesicles are the primary source of plasma S1P [11–15]. S1P mediates its biological function via five receptor subclasses (S1P1–5), where S1P1–3 are characteristic for the cardiovascular system influencing cardiac morphogenesis [16], endothelial integrity [17], smooth muscle cell function [18], and heart rate [19]. Overall, SP and S1P have a protective role in ischemia/reperfusion injury (IRI) in the heart [20]. Nevertheless, they are also involved in atherogenesis [21] and vessel remodeling [22]. Apart from metabolic function, S1P has a crucial position in bone marrow (BM) stem cell mobilization and homing [23]. Stem cell mobilization is S1P1- and S1P3-dependent [24, 25], while S1P2 receptor activation promotes BM cell retention [26].

The complement cascade consists of more than 50 proteins functionally associated with receptors and regulatory proteins. The mechanism of CC activation is based on cascade enzymatic cleavage of specific proteins [27]. Apart from its crucial role in innate and adaptive immune system response against pathogens, CC is involved in AMI- and CABG-induced inflammatory processes and stem cell mobilization [6, 28–30].

The study aimed at exploring further the role of BS and CC in the myocardial injury induced by AMI and cardiac surgery.

## 2. Patients and Methods

The study population consisted of 37 patients (mean age 57.8 ± 11.9 years) including 22 patients (59%) with ST-segment elevation myocardial infarction (STEMI) (2nd and 3rd Department of Cardiology, Medical University of Silesia) and 7 patients (19%) undergoing CABG (Department of Cardiac Surgery, Medical University of Silesia). Eight healthy individuals consisted a control group (CTRL). The study adhered to the principles of the Declaration of Helsinki and was approved by the Ethics Committee of the Medical University of Silesia in Katowice.

The project was funded by the European Union structural funds—Innovative Economy Operational Programme, Grant POIG.01.01.02-00-109/09 “Innovative methods of stem cells applications in medicine”, and statutory funds of Medical University of Silesia (KNW-2-052/D/5/N). Moreover, Tomasz Jadczyk was supported by the DoktoRIS—Scholarship Program for Innovative Silesia.

### 2.1. Patients

After hospital admission, medical history taking, and physical examination, individuals with AMI underwent coronary angiography with radial or femoral artery vascular access. Subsequently, pPCI with stent(s) implantation was performed on the infarct-related artery.

Exclusion criteria were as follows: (1) history of myocardial infarction within 30 days prior to study enrolment, (2) history of coronary artery intervention or CABG within 30 days prior to study enrolment, (3) pregnancy, (4) neoplasm, (5) chronic kidney failure (eGFR < 30 mL/kg/min), (6) liver failure, (7) coagulopathies and/or hematopoietic system diseases, (8) autoimmunological disorder, (9) systemic inflammatory process, (10) chronic obstructive pulmonary disease, (11) myopathies, and (12) muscle injury within 30 days prior to study enrolment.

Inclusion criteria were as follows: (1) age 18–80 years, (2) AMI diagnosed according to the European Society of Cardiology guidelines and referred for primary PCI within *<*12 hours after the onset of chest pain, or (3) multivessel, coronary artery diseases, referred for elective CABG, and (4) signed written informed consent.

### 2.2. Laboratory Investigations

In AMI patients, blood samples were obtained at hospital admission and 6, 12, 24, and 48 hours post pPCI. In the CABG group, blood samples were drawn 6, 12, 24, and 48 hours after cardiac surgery. In the control group, the samples were taken once. Samples (5 mL of peripheral blood) were drawn, mixed with anticoagulant (EDTA), and centrifuged within 1 h (10 min, 20°C; 2500 rpm). Plasma was divided into 3-4 tubes and stored in −20°C until analysis.

### 2.3. Plasma Concentration of CC Cleavage Fragments

Analysis of C3a, C5a, and C5b9 plasma concentrations was carried out using commercially available, highly sensitive ELISA kits (duplicate measurements): (1) C3a—Human C3a Platinum ELISA, BMS2089TEN, eBioscience; (2) C5a—Human C5a Platinum ELISA, DE3327, eBioscience; and (3) C5b9—Human Terminal Complement Complex C5b-9 (C5b-9) ELISA Kit, DL-C5b-9-Hu, DLDEVELOP, according to the manufacturer's protocol.

### 2.4. Plasma Concentration of Sphingosine and Sphingosine-1-Phosphate

SP and S1P plasma concentration measurements were performed as previously described [31, 32]. Briefly, plasma (300 *μ*L) was thawed at room temperature, and internal synthetic standard D-erytro-sphingosine-1-phosphate (S1P C18, Avanti Polar Lipids Company) and chloroform/methanol mixture (1 : 2, *v*/*v*) were added. Then, samples were sonicated for 10 minutes, and 600 *μ*L 1 M NaCl, 600 *μ*L chloroform, and 60 *μ*L 3 M NaOH were added. The samples were thoroughly vortexed and centrifuged (10 min, 5000 rpm). The upper aqueous phase containing sphingoid phosphates was transferred to another tube. The lower phase was extracted (with 600 *μ*L 1 M NaCl, 600 *μ*L chloroform, and 60 *μ*L 3 M NaOH), mixed, and centrifuged once more. Both aqueous phases were combined, and chloroform (1500 *μ*L) and concentrated HCl (160 *μ*L) were added. The samples were vortexed and centrifuged (10 min, 5000 rpm). The lower organic phase was transferred to another tube and vacuum-dried in a SpeedVac for 45 minutes in 45°C (RVC 2-25 CD, Martin Christ GmbH) and stored at −80°C until assays were performed.

Directly before analytical measurements, dried residue was dissolved in methanol (150 *μ*L). After addition of reaction mixture OPA to derivatization (5 mg *o*-phthalaldehyde, 100 *μ*L methanol, 5 *μ*L of mercaptoethanol, and 5 mL of boric acid in pH 10.5), samples were incubated at room temperature and then centrifuged (10 min, 5000 rpm). The clear supernatant was transferred to a fresh tube and subjected to RP HPLC analysis.

Analysis of S1P was carried out using a Hewlett Packard Series 1200 (Agilent, USA). Reversed-phase HPLC was performed on the column Cosmosil 5 *μ*m C18-ARII (150 × 4.6 mm) with precolumn 5 *μ*m C18-ARII (10 × 4.6 mm) (Waters). The column temperature amounted to 25°C. The isocratic method with active phase consisting of 10 mM K_2_HPO_4_ (pH 5.5) and methanol (15 : 85; *v*/*v*) was applied. The flow rate was 1.0 mL/min. 50 *μ*L samples were injected every 30 minutes. The wavelength for detection of the derivatives of S1P was 340 nm for excitation and 455 nm for emission. The quantitation was based on peak areas with and without internal standard calibration (S1P-C17 from Avanti Polar Lipids).

### 2.5. Plasma Concentration of Free Hemoglobin

The concentration of free hemoglobin was assessed applying spectrophotometry (UV/VIS Lambda 650, PerkinElmer, USA) with Drabkin's reagent [33].

### 2.6. Statistical Analysis

Statistical analysis was carried out using SAS University Edition (SAS Institute Inc., Cary, NC, USA) and expressed as the mean ± standard deviation (SD). Qualitative data were expressed as crude values and/or percentages. Differences between groups were analyzed using *T*-test for normally distributed data and Mann–Whitney *U* test for nonnormally distributed data. Within-group plasma concentration differences of analyzed factors were assessed using the one-way analysis of variance (ANOVA) test for normally distributed data and Friedman Two-way Analysis of Variance by Ranks test for nonnormally distributed data. A post hoc group comparison was performed with Tukey's and Dunn's test, as appropriate. Data distribution was verified with the Shapiro-Wilk test [34]. A value of *p* < 0.05 was considered as significant.

## 3. Results

Clinical characteristics of the study population are presented in Table 1. In the CABG group, the number of patients with hypertension, dyslipidemia, and diabetes mellitus was significantly higher than in the AMI group. Moreover, there were a numerically higher number of males in all groups. All patients in the AMI group were treated successfully with TIMI 3 flow in the culprit vessel. No deaths and adverse events were noted during the study.

In the CABG group, surgery and the periprocedural period were uncomplicated. Patients healed their sternal wounds uneventfully without subsequent problems allowing timely respiratory and mobility rehabilitation.

### 3.1. Complement Cascade Cleavage Fragments

**Figure 1:**
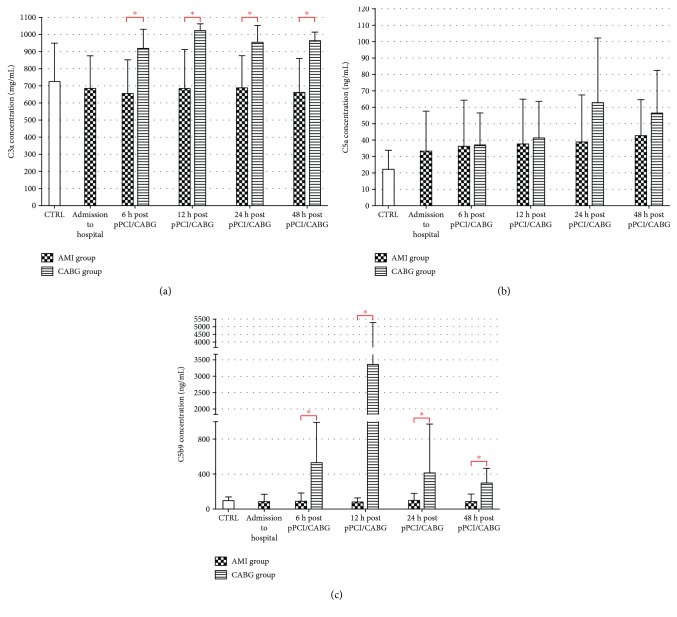
CC component (a) C3a, (b) C5a, and (c) C5b9 plasma concentration in AMI, CABG, and CTRL (^∗^*p* < 0.05). AMI: acute myocardial infarction; CABG: coronary-artery bypass graft; CTRL: control group; pPCI: primary percutaneous coronary intervention.

Within the 48-hour observational period, C3a and C5b9 plasma levels were significantly higher in the CABG group when compared to AMI patients (*p* < 0.01). Also, levels of C3a were higher in CABG than in the CTRL group. In patients with MI, C5a concentration was 2-fold higher in comparison with the control group at 12 and 48 h hours after admission (Figure 1).

**Figure 2:**
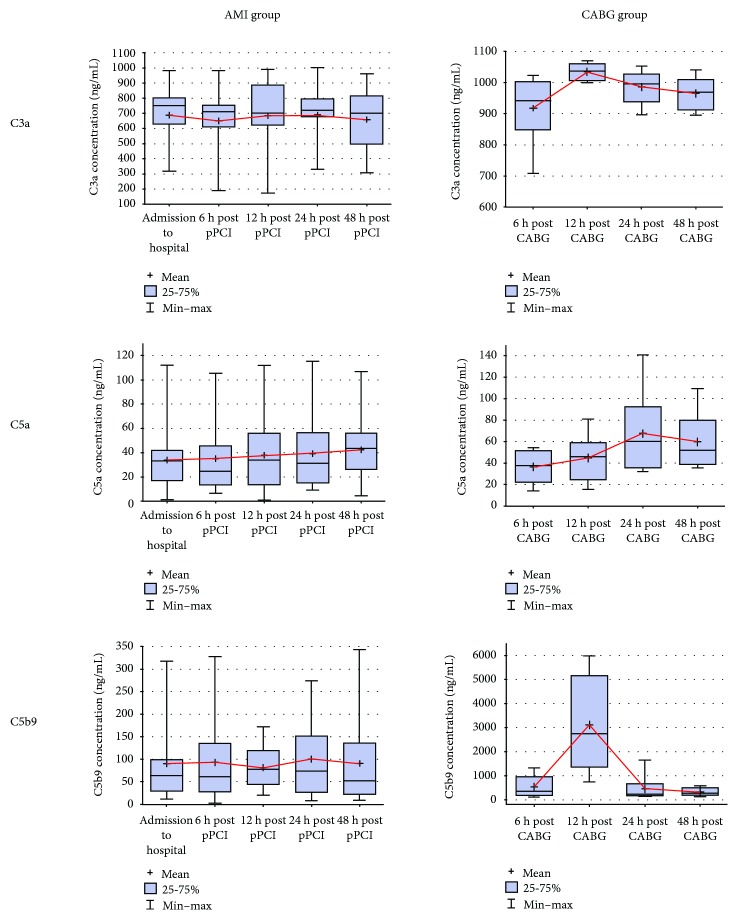
Dynamics of C3a, C5a, and C5b9 plasma concentrations in AMI and CABG patients within 48 hours post-pPCI/CABG. AMI: acute myocardial infarction; CABG: coronary-artery bypass graft; pPCI: primary percutaneous coronary intervention.

A point-by-point analysis of CC component plasma concentrations within 48 hours did not show statistically significant differences between AMI and CABG patients (Figure 2).

### 3.2. Sphingosine and Sphingosine-1-Phosphate

**Figure 3:**
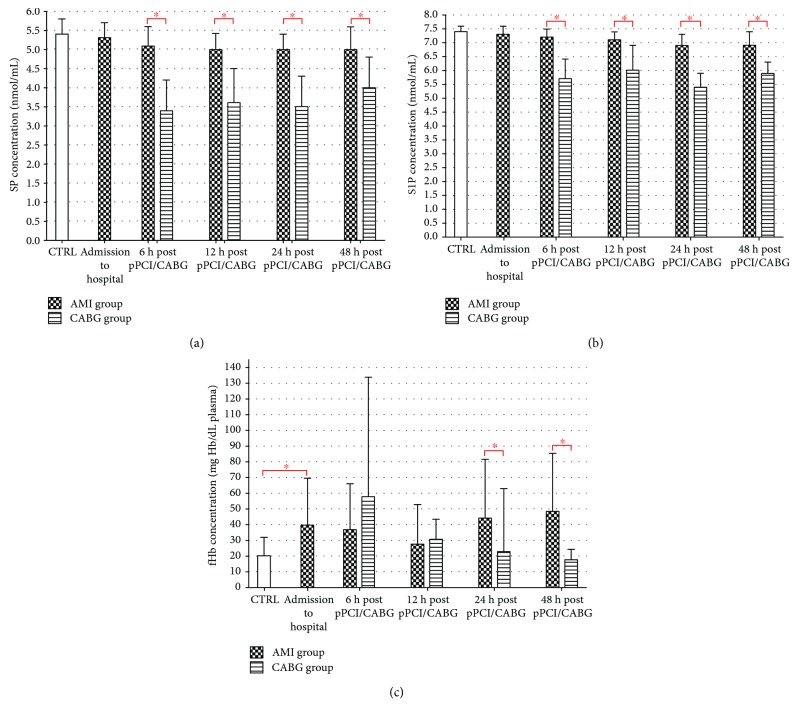
(a) SP, (b) S1P, and (c) fHb plasma concentrations in AMI, CABG, and CTRL (^∗^*p* < 0.05). AMI: acute myocardial infarction; CABG: coronary-artery bypass graft; CTRL: control group; fHb: free hemoglobin; pPCI: primary percutaneous coronary intervention; S1P: sphingosine-1-phosphate; SP: sphingosine.

**Figure 4:**
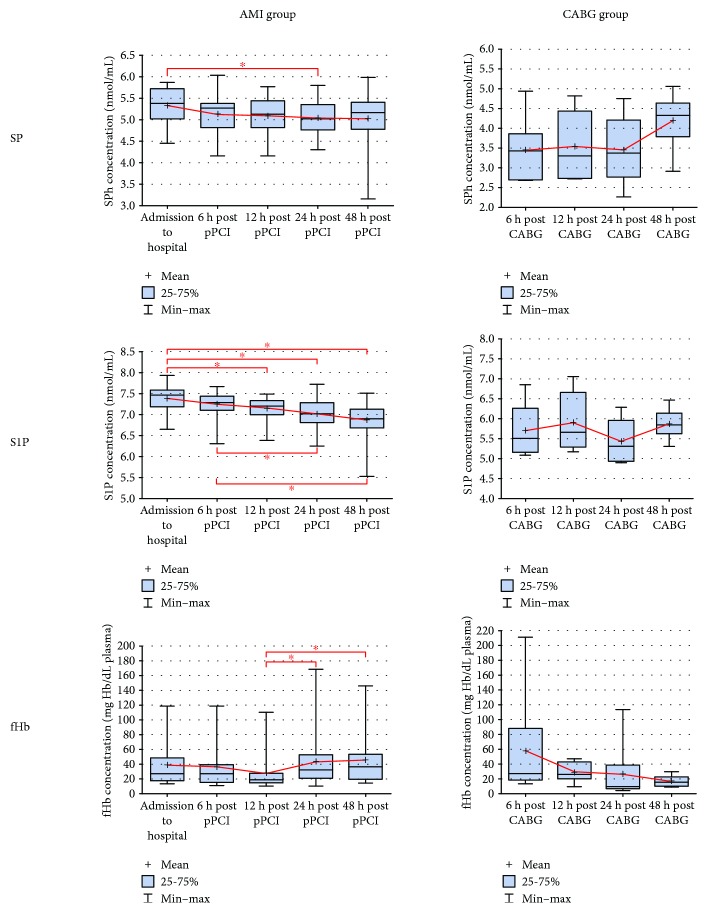
Dynamics of SP, S1P, and fHb plasma concentrations in AMI and CABG patients within 48 hours post pPCI/CABG. AMI: acute myocardial infarction; CABG: coronary-artery bypass graft; fHb: free hemoglobin; S1P: sphingosine-1-phosphate; SP: sphingosine, ^∗^*p* < 0.05.

Oppositely, SP and S1P concentrations were significantly lower in subjects undergoing CABG compared to AMI patients (*p* < 0.01). Moreover, in comparison to CTRL, BS showed similar levels in CABG and AMI groups (Figures3(a)[SP] and3(b)[S1P]). In patients with myocardial infarction, there is a significant decrease in S1P and SP levels starting from 12 and 24 hours post pPCI, respectively (Figure 4).

### 3.3. Free Hemoglobin

The plasma concentration of fHb was significantly higher in patients with acute MI in comparison to the CABG group 24–48 hours post pPCI. Furthermore, at admission and 24 and 48 hours after AMI, there was a ~2–2.5 fold increase in fHb level compared to CTRL (Figure 3(c)). Moreover, in the AMI group, fHb concentration showed an increase between 12 h and 24–48 h time points after stent implantation (Figure 4).

## 4. Discussion

There are inconsistent data regarding CC and BS function in ischemic myocardium. Thus, the current study assesses dynamics of plasma C3a, C5a, C5b9, SP, S1P, and fHb concentration changes in post-pPCI AMI patients and stable CAD subjects undergoing CABG procedure. Both clinical scenarios are associated with a pathogen-free intense inflammatory process initiated by ischemia/reperfusion and mechanical trauma [4]. Correspondingly to microbial-induced inflammation, so-called sterile inflammation is characterized by infiltration of neutrophils and synthesis of cytokines/chemokines (i.e., tumour necrosis factor and interleukin-1 [IL-1]) [35]. However, distinct triggers, activation, and signaling pathways are also involved in this form of inflammatory response. A multidirected activation of proteolytic enzyme pathways (complement cascade, coagulation, and fibrinolysis system) occurs with the secretion of molecular agents termed danger-associated molecular patterns (DAMPs). Subsequently, the signal is transduced via specialized receptors, such as toll-like receptors (TLRs) and the NOD-like receptor family, inducing upregulation of IL-1*β*, which promotes recruitment of other inflammatory cells [35].

Regarding the presented study, it is important to note that it does not directly address an association between sterile inflammation and changes of BS, CC, and fHb levels. Dynamics of plasma concentration is presented in a context of literature data strongly supporting involvement of analyzed particles in the myocardium injury-induced process.

The complement cascade is a crucial element of the adaptive and innate immune system [36]. Apart from antimicrobial activity, CC is a part of the DAMP recognition system allowing tissue clearance under pathological conditions including myocardial infarction and general inflammatory response in patients undergoing CABG procedure [7, 37]. Moreover, it plays an important role in the pathogenesis of coronary artery disease being associated with plaque instability and a higher risk of vascular complications [38]. In patients with AMI, the plasma level of CC components is a resultant of intravascular cascade activation (induced by plaque rupture and thrombus formation) [39] as well as intramyocardial CC induction [40]. Importantly, complement activation is also associated with a percutaneous coronary intervention, a first-line treatment strategy in patients with STEMI. Recent studies investigating dynamics of plasma CC component concentration in post-pPCI AMI patients showed heterogeneous results [41–45]. In the analysis by Karapetyan et al., an elevated C5b9 level was observed 24 h after pPCI [44], whereas, within the same timeframe, the research group of Horváth described an opposite result [45]. Furthermore, Orn et al. described the statistically significant increase in plasma C5b9 within 7 days post AMI with subsequent normalization after 2 months [41]. Oppositely, Cubedo et al. found reduced C3 concentration after 72 h post pPCI [43].

As mentioned, in patients with AMI complement, cascade activation is triggered during intravascular thrombus formation. Crucially, there is a direct interaction between CC, coagulation, and fibrinolytic cascade components, as well as platelets. This fact is important regarding study results because both thrombin and plasmin have C5 convertase-like activity [29, 46]. C5 plays a pivotal role in an acute phase of myocardial infarction being associated with augmentation of the inflammatory process and tissue damage. C5 is a strong chemotactic factor for neutrophils increasing their adhesion to endothelium [47, 48], stimulating them to produce reactive oxygen species (ROS) and proteolytic enzymes [49]. Moreover, IRI-induced C5a component generation is involved in the synthesis of cytokines, chemokines, and proinflammatory molecules [50, 51]. Regarding myocardial function, a complement cascade is predominantly presented in a negative and deleterious context [52, 53]. Nevertheless, recent studies cast new light on CC showing its role in the regeneration process through cell growth and differentiation, antiapoptotic activity, and bone marrow stem/progenitor cell (BMSPC) mobilization [54]. In contrast to C5, C3 has a dominant role in a chronic phase of myocardial infarction. C3 deficiency due to gene deletion results in left ventricle dysfunction, remodeling, and dilatation. Moreover, in the animal model, pathological consequences were related to reduced number and proliferation potential of c-kit+ cardiac stem/progenitor cells as well as impaired BMSPC mobilization [54].

Correspondingly, CABG surgery triggers an intensive local and general inflammatory response activating CC, coagulation, and fibrinolytic cascade. Dynamic changes of complement function during this procedure are associated with surgical trauma, bioincompatibility of the cardiopulmonary bypass circuit (CBP), pharmacotherapy (general anesthesia, heparin, and protamine), and IRI [6, 55]. Cardiac surgery initiates complement classical and lectin pathways [37, 56]. Furthermore, operational tissue injury is responsible for the synthesis of plasmin, which has C3 and C5 convertase-like activity [29, 57]. Importantly, heparinization of CBP improves biocompatibility [58, 59] reducing C3 surface adsorption, thus inhibiting the alternative pathway [60, 61]. Furthermore, heparin impairs coagulation system function decreasing thrombin formation which has C5 convertase-like activity [29]. Interestingly, reversal of heparinization by protamine application alters CC through classical pathway activation [62].

Hoedemaekers et al., analyzing CC function during postoperative period, showed a biphasic activation pattern of the classic pathway (reduction within the first 8 h after CABG with subsequent increase). Simultaneously, researchers described monophasic alternative pathway deactivation. It is worth noting that, in a small population of patients, a biphasic pattern of classic pathway activity was not seen [56].

In the current study, when compared to AMI patients, a higher CC cleavage fragment concentration in the CABG group might be resultant of general anesthesia, periprocedural tissue damage, and/or extracorporeal circulation. Postoperative C3a concentration changes correspond to a monophasic dynamics described by Hoedemaekers et al. [56]. Oppositely, the C5b9 plasma level showed different characteristics. Interestingly, there was an increase in C5b9 concentration without increase in C5a in the same timeframe. This result, as hypothesis [56], indicates that CC activity is decreased at the level of C5 convertase. Moreover, changes in C5b9 and fHb plasma levels have a biphasic pattern with an initial increase in concentration within 6–12 h after surgery and subsequent decrease.

SP and S1P are responsible for fundamental cellular functions playing an important role in physiology and pathophysiology of the cardiovascular system [63]. S1P synthesis in myocardium mediates a cardioprotective function against cell damages caused by IRI [64]. Noteworthy, short-term ischemia triggers generation of ROS, which induces sphingosine kinase (SK) activity resulting in an increased tissue S1P concentration [65]. Conversely, long-lasting ischemia is associated with SK degradation [66], which could explain the dynamics of S1P plasma concentration changes observed in this study. SP and S1P concentrations decreased in AMI patients within 48 hours after pPCI, which corresponds to S1P dynamics described by Knapp et al. [67]. Similarly, the detailed lipidomic analysis in patients with acute coronary syndrome showed the statistically significant lower concentration of S1P in comparison with healthy individuals [68]. On the contrary, Karapetyan et al. described higher—compared to the control group—level of this phosphosphingolipid in STEMI patients during the 48-hour observation period [44].

According to the best knowledge of the authors, the presented experiment is the first study evaluating the dynamics of bioactive sphingolipid plasma concentration in patients undergoing CABG surgery. Interestingly, in this group, there is a statistically significant lower SP and S1P concentration in comparison with AMI patients and control. The results might be related to systemic metabolic response as well as changes in the activity of enzymes involved in sphingolipid metabolism. In this context, the study findings could be explained by the decreased activity of SK1 and SK2 or increased activity of S1P phosphatase and lyase. Furthermore, restrictive diet preceding surgical procedure and mechanical ventilation during the surgery could influence BS metabolism. Sun et al. proved that hypoxia induces higher SK1 activity [69]. On the contrary, in patients undergoing CABG, periprocedural oxygenation could reduce SK activity decreasing S1P concentration. It must also be noted that the evaluation of plasma sphingolipid level was analyzed without assessment of myocardial tissue concentration, which could impact a final result.

Limitations of this study include the small number of patients. A larger study would be needed to more clearly understand the dynamics of SP, S1P, C3a, C5a, C5b9, and fHb.

Moreover, in the case of sphingolipids, appropriate sample collection and preparation are critical to obtaining reliable results. In the current study, after taking blood from a peripheral vein, samples were kept on ice to avoid complement cascade activation and release of sphingolipids from platelets. On the other hand, in vivo, S1P has a short half-life (15 min) suggesting a dynamic metabolism. Thus, it would be more preferable to maximally reduce time between sample collection, centrifugation, and storage. In the study, despite utilization of the ice, samples were proceeded within 1 hour, which could influence the results. Moreover, it is important to note that centrifugation parameters are very important. Frej et al. [70] compared the effect of centrifugation speed and time (300*g* for 15 min versus 1000*g* for 10 min versus 2000*g* for 10 min versus 2000*g* for 20 min) on platelet count and S1P concentration establishing the most optimal protocol (2000 g for 10 min). In the presented study, samples were vortexed and centrifuged at 5000 rpm for 10 min to obtain a platelet-free plasma. This strategy was also applied in the study by Knapp et al. [71], who analyzed the dose-dependent effect of aspirin on plasma sphingolipid levels.

In regard to study limitations, the presented hypotheses require further detailed experimental investigation.

The knowledge about bioactive sphingolipids and complement system components may help to optimize therapeutic strategy in patients with myocardial infarction and individuals undergoing CABG procedure. Novel pharmacological agents such as fingolimod (FTY720) or amiseliod are promising candidates [72].

## 5. Conclusion

Plasma levels of BS and CC cleavage fragments are significantly different in AMI and CABG patients. Post-pPCI, SP, and S1P concentrations were higher in comparison to individuals undergoing a surgical procedure. Conversely, C3a and C5b9 plasma concentrations were higher in the CABG group. Moreover, the dynamics of analyzed compounds was different between the groups. In AMI patients, S1P and SP concentration decrease was observed after 12 and 24 h post pPCI, respectively. Moreover, 1 and 2 days after percutaneous revascularization, fHb plasma level was significantly higher in comparison to patients undergoing CABG.

## Figures and Tables

**Table 1:** Baseline characteristics of study groups.

	AMI group *n* = 22	CABG group *n* = 7	CTRL group *n* = 8
Males, *n* (%)	17 (77.3)	6 (85.7)	4 (50.0)
Age (mean ± SD)	58.4±12.7	55.6 ± 9.3	48.1 ± 4.9
Previous myocardial infarction, *n* (%)	4 (18.2)	1 (14.3)	0 (0)
Previous percutaneous coronary intervention, *n* (%)	3 (13.6)	1 (14.3)	0 (0)
Previous coronary-artery bypass graft surgery, *n* (%)	1 (4.5)	0 (0)	0 (0)
Hypertension, *n* (%)	14 (63.6)	7 (100)	0 (0)
Diabetes mellitus, *n* (%)	3 (13.6)	3 (42.9)	0 (0)
Dyslipidemia, *n* (%)	11 (50.0)	7 (100)	0 (0)
Current smoking, *n* (%)	12 (54.5)	6 (85.7)	1 (12.5)
Family history of cardiovascular diseases, *n* (%)	8 (38.1)	3 (75.0)	1 (12.5)

AMI: acute myocardial infarction; CABG: coronary artery bypass graft; CTRL: control group; SD: standard deviation.
